# Genome-wide association study identifies single-nucleotide polymorphism in *KCNB1 *associated with left ventricular mass in humans: The HyperGEN Study

**DOI:** 10.1186/1471-2350-10-43

**Published:** 2009-05-19

**Authors:** Donna K Arnett, Na Li, Weihong Tang, Dabeeru C Rao, Richard B Devereux, Steven A Claas, Rachel Kraemer, Ulrich Broeckel

**Affiliations:** 1 Department of Epidemiology, School of Public Health, University of Alabama at Birmingham 1530 3rd Avenue South, Birmingham AL 35294-0022, USA; 2 Division of Biostatistics, University of Minnesota, A460 Mayo Building, 420 Delaware St SE, Minneapolis, MN 55455, USA; 3 Division of Epidemiology and Community Health, University of Minnesota, West Bank Office Building, 1300 S. Second Street, Suite 300, Minneapolis, MN 55454-1015, USA; 4 Division of Biostatistics, Washington University in St. Louis, 660 South Euclid Avenue, Box 8067, St Louis, MO 63110-1093, USA; 5 Division of Cardiology, Weill Medical College of Cornell University, Greenberg Division of Cardiology, 520 East 70th Street, 4th Floor, New York, NY 10021, USA; 6 Department of Medicine, Medical College of Wisconsin, 8701 Watertown Plank Road, Milwaukee, WI 53226, USA

## Abstract

**Background:**

We conducted a genome-wide association study (GWAS) and validation study for left ventricular (LV) mass in the Family Blood Pressure Program – HyperGEN population. LV mass is a sensitive predictor of cardiovascular mortality and morbidity in all genders, races, and ages. Polymorphisms of candidate genes in diverse pathways have been associated with LV mass. However, subsequent studies have often failed to replicate these associations. Genome-wide association studies have unprecedented power to identify potential genes with modest effects on left LV mass. We describe here a GWAS for LV mass in Caucasians using the Affymetrix GeneChip Human Mapping 100 k Set. Cases (N = 101) and controls (N = 101) were selected from extreme tails of the LV mass index distribution from 906 individuals in the HyperGEN study. Eleven of 12 promising (*Q *< 0.8) single-nucleotide polymorphisms (SNPs) from the genome-wide study were successfully genotyped using quantitative real time PCR in a validation study.

**Results:**

Despite the relatively small sample, we identified 12 promising SNPs in the GWAS. Eleven SNPs were successfully genotyped in the validation study of 704 Caucasians and 1467 African Americans; 5 SNPs on chromosomes 5, 12, and 20 were significantly (*P *≤ 0.05) associated with LV mass after correction for multiple testing. One SNP (rs756529) is intragenic within *KCNB1*, which is dephosphorylated by calcineurin, a previously reported candidate gene for LV hypertrophy within this population.

**Conclusion:**

These findings suggest *KCNB1 *may be involved in the development of LV hypertrophy in humans.

## Background

Increased mass of the left ventricle (LV) is considered a compensatory process which maintains cardiac output in response to pathological stimuli such as hypertension, obesity, and myocardial injury [1,2]. Left ventricular mass is a sensitive predictor of cardiovascular mortality and morbidity in all genders, races, and ages [3-6]. Evidence suggests LV mass is, in part, under genetic control, with heritability estimates generally falling between 0.17 and 0.59 [7-10]. The normal distribution of LV mass in populations suggests this phenotype is a complex trait influenced by multiple genes. Some studies have reported statistically significant association between LV mass and the angiotensin converting enzyme gene (*ACE*) [11], guanine nucleotide-binding protein gene (*GNB3*) [12], insulin-like growth factor gene (*IGF-1*) [13], and neuropeptide Y gene (*NPY*) [14], although other studies have failed to provide evidence of association of LV mass with some of these same genes (e.g., *ACE *[15], *GNB3 *[16]). Although the dearth of consonant findings may be more indicative of the nature of complex diseases than the quality of the conflicting studies, candidate gene association studies are, by definition, limited to investigating genes with previously known connections to the phenotype of interest. Linkage studies have the advantage of scanning the entire genome without such assumptions; however, they are underpowered relative to association studies for common, complex genetic diseases [17]. Genome-wide association studies (GWAS) offer both the exploratory benefits of linkage and the statistical power of association.

We describe here a GWAS for LV mass in Caucasians using the Affymetrix GeneChip Human Mapping 100 k Set followed by a candidate SNP validation study. Cases and controls were selected from the extremes of the LV mass index distribution from 906 individuals in the Family Blood Pressure Program (FBPP) – Hypertension Genetic Epidemiology Network (HyperGEN) Study. The most significant SNPs identified in the GWAS (i.e., those with the smallest raw *P *values) were examined in a validation study of 704 Caucasians and 1467 African Americans. Five SNPs on chromosomes 5, 12, and 20 were significantly associated with LV mass in the validation study. One SNP (rs756529) is intragenic within *KCNB1*, which is dephosphorylated by calcineurin, a previously reported candidate gene for LV hypertrophy within this population [18].

## Methods

### Study population

Study participants (101 cases, 101 controls for the GWAS; 2171 individuals for validation) were recruited from the FBBP – HyperGEN Study [19]. HyperGEN is one of four networks initiated and supported by the National Heart, Lung and Blood Institute to identify genetic contributions to hypertension. Subjects were drawn from population-based cohorts (from the Atherosclerosis Risk in Communities Study in Minneapolis, MN and Forsyth County, NC; the Minnesota Heart Survey; and the Utah Health Family Tree Study), or from the community-at-large (Birmingham, AL). Recruitment criteria required that participating sibships had ≥ 2 siblings who had been diagnosed with hypertension before age 60. Hypertension was defined as currently taking antihypertensive medication or having an average systolic blood pressure ≥ 140 mm Hg and/or diastolic blood pressure ≥ 90 mm Hg measured at two separate clinic visits. Average blood pressure was calculated using the second and third measurements of three readings using an oscillometric blood pressure monitor (Dinamap 1846 SX/P; GE Healthcare, Waukesha, WI, USA). Individuals with a history of type 1 diabetes or severe renal disease were excluded. As this was a pilot GWAS, we selected a modest number of cases and controls from the Caucasian strata in a manner to maximize our detection of association. Because we had observed linkage evidence for the LV mass index (LVMI, defined as LV mass/height^2.7^) phenotype (linkage data published separately [20]) in the hypertension-affected sibships of the HyperGEN population [21], we defined cases as subjects in the extreme of the log-transformed (and roughly normal) LVMI distribution. Specifically, cases with the largest LVMI values were selected in descending order from the largest value. The reverse process was used to select controls: individuals were selected from smallest LVMI values in ascending order from the smallest value. Thus, cases and controls were those above and below approximately the 90^th ^and 10^th ^percentile of the LVMI distribution, respectively. The validation study samples consisted of all remaining (i.e., after case-control selection) 704 Caucasian siblings and all African American hypertensive siblings (N = 1467) participating in the HyperGEN study. For comparison purposes, a validation study association analysis was also conducted with the combined (i.e., the 202 individuals used as cases and controls plus the remaining 704 individuals, N = 906) Caucasian sample. About 85% of the African Americans were recruited from geographically distant center (i.e., Birmingham, AL) from the Caucasian recruitment centers (e.g., Utah, Minnesota, and North Carolina), and to that end, serve as a distinct population for the purposes of replication. This study was approved by the centers' institutional review boards, and all subjects gave informed consent.

### Echocardiographic measures

Doppler, two-dimensional (2D), and M-mode (2D-guided) echocardiograms were performed following a standardized protocol previously described [22]. Certified sonographers from each field center were trained at the echocardiography reading center (New York Hospital-Cornell Medical Center). M-mode and 2D echocardiograms via the parasternal acoustic window were recorded for ≥ 10 beats. Measurements were made at the echocardiography reading center using a computerized review station equipped with a digitizing tablet and monitor overlay used for calibration and quantification (Digisonics, Inc., Houston, TX, USA). LV linear dimensions were measured by M-mode or 2D echocardiography according to American Society of Echocardiography recommendations [23,24]. LV mass was calculated using end-diastolic dimensions by an anatomically validated formula [25], and indexed to height^2.7 ^as described above. The reproducibility of echocardiographic measures was assessed in a substudy of 12 individuals who had echocardiograms conducted two weeks apart.

### Genotyping methods

The Affymetrix GeneChip Human Mapping 100 k Set, consisting of two assays and two microarrays, was used for the genome-wide scan. Methods were adapted from Matsuzaki et al. [26]. All genotyping results were analyzed using the Affymetrix analysis software (GDAD and GTYPE). Duplicate SNPs were typed on both chips in order to detect potential sample mix-ups. For the validation study, SNPs found to be significant in the GWAS were genotyped using the Applied Biosystems (ABI, Foster City, CA, USA) TaqMan technology; probes were designed using either Assay-on-Demand or Assay-by-Design.

### Statistical and analytical methods

For the GWAS analysis, we first screened out monomorphic markers and markers that were not likely to be informative as defined by minor allele frequency < 0.05. A simple logistic model was used to analyze the association of case-control status with each SNP separately. Since some of the subjects were siblings (a total of 55 in cases and controls), empirical standard error estimates were used to correct for the within-family correlation using generalized estimation equations (GEE) with exchangeable working correlation matrix [27,28]. Because the number of minor allele homozygotes in our sample tended to be small (e.g., ~8 for a SNP with a minor allele frequency of 20%), we chose to use a dominant model. Adjusted *P *values were calculated using Holm's method to control for family-wise error rate [29]. *Q *values [30] were calculated using the qvalue package in R to allow control of the false discovery rate. All analyses for the GWAS were carried out using R .

Eleven of the 12 chosen SNPs from the GWAS could be successfully genotyped for the two ethnic groups in the validation study. For the validation study SNPs, tests of genotype distribution for Hardy-Weinberg equilibrium were conducted in each race group by an exact test implemented in the computer program Pedstats [31]. HWE was examined in all individuals and a set of unrelated individuals (876 African Americans and 545 Caucasians) that were selected by an algorithm implemented in Pedstats. Since multiple SNPs were tested, *P *< 0.01 was considered to be statistically significant for violation of HWE.

Two phenotypes were examined in the validation study, the continuous variable of LVMI and the categorical phenotype of left ventricular hypertrophy (LVH) defined as LVMI > 47 g/m^2.7 ^in women or > 50 g/m^2.7 ^in men. We used GEE with an exchangeable working correlation matrix for within-family correlation to test the association between the validation study SNPs and continuous and categorical LV phenotypes [27,28]. Covariates that were adjusted for included age, sex, weight, systolic blood pressure, and diabetes. For each SNP, genotypes were grouped into two categories in the same manner as in the GWAS analysis. Natural log-transformation was performed for LVMI to normalize the trait distribution. As noted above, for Caucasians we also analyzed the GWAS and validation study participants in a combined analysis.

## Results

In the echocardiographic reproducibility substudy, the intraclass correlation coefficient was 0.90. In another study using methods comparable to those of HyperGEN, the echocardiography reading center at Cornell reported good reproducibility of LV mass (r_i _= 0.93) [32]. All Affymetrix GeneChips passed the standard cut-off for the genotype call rate (< 85%). Approximately 8% of the SNPs on the gene chip set were monomorphic in our sample; another ~10% had minor allele frequencies less than 5%, leaving 96,258 informative SNPs in our genome-wide scan.

In the GWAS, both the case and control samples had mean ages of about 60 years and women comprised about 66% of both groups. The case group had a significantly higher mean body mass index, systolic blood pressure, pulse pressure, and, by design, LVMI. Mean LVMI in cases was 56.2 ± 6.3 g/m^2.7^; in controls mean LVMI was 31.4 ± 3.9 g/m^2.7^. Other demographic and clinical characteristics of cases and controls are shown in Table 1.

**Table 1:** Demographic and clinical characteristics by case-control status and validation study population.

	**Genome-wide association**			**Validation study**	
		
**Characteristics**	**Case** **(N = 101)**	**Control** **(N = 101)**	P*	**Caucasians (N = 704)**	**African Americans** **(N = 1467)**
Age, years	59.1 ± 9.1	60.3 ± 9.3	0.34	60.7 ± 8.34	50.8 ± 11.0
Females, %	66.3	66.3	1.00	48.9	64.4
Diabetic, %	19.8	12.9	0.18	15.8	22.8
Weight, kg^†^	90.1 (70.9 – 114.4)	79.6 (64.2 – 98.7)	0.0001	85.5 ± 17.8	89.9 ± 21.5
Height, cm	164.6 ± 8.0	169.0 ± 8.7	0.0002	168.9 ± 9.1	167.3 ± 9.1
Body mass index, kg/m^2†^	33.4 (26.7 – 41.8)	27.9 (23.3 – 33.5)	< 0.0001	29.9 ± 5.5	32.2 ± 7.5
Percentage of body fat^‡^	39.3 ± 8.9	36.0 ± 8.2	0.012	35.3 ± 8.6	39.9 ± 9.5
Systolic blood pressure, mm Hg	135.5 ± 20.8	126.1 ± 15.7	0.0003	126.8 ± 20.2	132.1 ± 22.5
Diastolic blood pressure, mm Hg	70.9 ± 12.5	71.0 ± 10.2	0.90	70.1 ± 10.6	75.2 ± 11.7
Pulse pressure, mm Hg	64.7 ± 16.2	55.1 ± 13.7	< 0.0001	56.7 ± 16.2	56.9 ± 16.7
Postural SBP change, mm Hg	-1.32 ± 14.7	-0.91 ± 15.0	0.85	-2.55 ± 14.8	1.63 ± 14.2
*Hypertension status*					
Normal BP, %	0.0	0.0	-	22.9	13.5
Mild hypertension, %	42.6	57.4	-	35.2	37.4
Severe hypertension, %	57.4	42.6	-	41.9	49.1
Antihypertensive drugs, N	1.43 ± 0.84	1.35 ± 0.71	0.42	1.38 ± 0.80	1.35 ± 0.92
LV mass, g	214.5 ± 32.8	130.3 ± 21.8	< 0.0001	171.7 ± 40.5	178.1 ± 49.8
LV mass/body surface area, g/m^2^	108.9 ± 15.0	68.0 ± 9.0	< 0.0001	87.6 ± 17.3	89.9 ± 22.7
LV mass/height^2.7^, g/m^2.7^	56.2 ± 6.3	31.4 ± 3.9	< 0.0001	41.6 ± 8.66	44.4 ± 12.1
Urinary albumin:creatinine ratio^†^	6.87 (1.93 – 24.4)	3.11 (0.58 – 16.6)	0.0002	3.74 (0.92 – 15.2)	7.24 (1.27 – 41.3)

*P for comparison of means between cases and controls; ^†^Reported as geometric mean (SD range) because natural-log transformed values were compared between cases and controls; ^‡^Lukaski equation; SBP = systolic blood pressure

We identified the 12 SNPs from the genome-wide analysis with the smallest raw *P *values to genotype in the subsequent validation study. The 12 SNPs all had unadjusted *P *values < 0.0001 and all had *Q *values < 0.8 [30]. The estimated positive false discovery rate was 75% [30,33]. The next best SNP (i.e., the SNP with the 13^th ^lowest raw *P *value) had a *Q *value of 1.00. The SNP with the strongest association in the genome-wide analysis (rs409045 on chromosome 5p13.2) had an adjusted *P *= 0.08 using Holm's method [29]. Table 2 lists unadjusted *P *values for the 12 top-ranked SNPs from the genome-wide scan.

**Table 2:** SNPs from the genome-wide scan with *Q *values < 0.8 (i.e., false-discovery rate < 80%).

**SNP**	**Chr**	Position*	**MAF**	***P***^†^
rs409045	5	34664384	0.44	8.47E-07
rs6450415	5	56384642	0.56	6.43E-05
rs1833534	5	162753563	0.19	9.62E-05
rs6961069	7	80056897	0.57	7.34E-05
rs10499859	7	80096746	0.69	3.27E-06
rs4129000	12	64239801	0.28	2.26E-05
rs4129218	12	64244928	0.38	5.19E-05
rs1155635	13	69845453	0.60	9.09E-05
rs2415872	14	44177211	0.72	8.68E-05
rs238688	20	3473701	0.33	8.33E-05
rs756529	20	47444415	0.68	9.10E-05
rs10483186	22	33669852	0.69	3.12E-05

*dbSNP positions are as bases, from NCBI Build 36; ^†^unadjusted *P *value; Chr = chromosome; MAF = Overall frequency of carriers of minor allele (homozygotes + heterozygotes); SNP = single-nucleotide polymorphism

**Figure 1:**
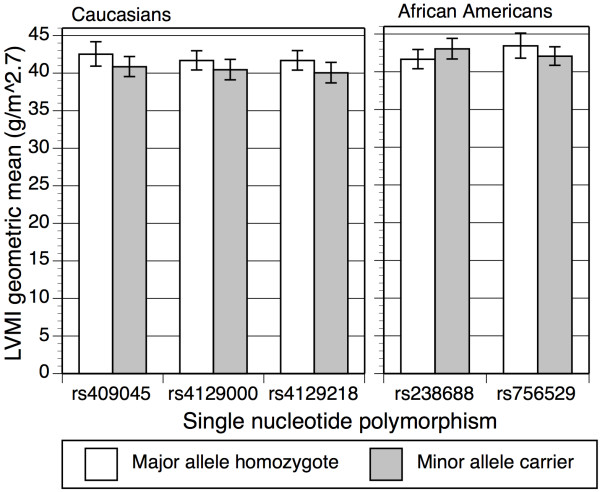
**Effect sizes (geometric means) of significant (*P *< 0.05) left ventricular mass index associations in the validation study for Caucasians and African Americans**. Error bars indicate 95% confidence intervals.

Demographic and clinical characteristics of the validation study samples are shown in Table 1. In African Americans, only one SNP (rs6961069) was not at HWE at *P *< 0.01. In Caucasians, no significant departures from HWE were detected for SNPs in the set of unrelated individuals. In the Caucasian replication sample, all but one of the SNPs identified by GWAS (rs756529) had minor alleles that were the same as those in the GWAS sample. In the African American validation study sample, six SNPs had different minor alleles than those in the Caucasian GWAS sample: rs6961069, rs10499859, rs756529, rs10483186, rs6450415, and rs4129418. Tables 3, 4, and 5 present the *P *values for the 11 successfully genotyped SNPs for the 704 Caucasians not used in the case-control GWAS, for the combined 906 Caucasian sample (i.e., 202 cases and controls plus 704 remaining Caucasians), and for the 1467 African Americans. Effect sizes, as geometric means, for significant associations (*P *< 0.05) with LVMI are shown in Figure 1. The effect sizes, as odds ratios, for the two significant (*P *< 0.05) associations with LVH were 1.60 (1.01, 2.52, 95% confidence interval) for rs4129218 in Caucasians and 1.40 (1.03, 1.92, 95% confidence interval) for rs756529 in African Americans.

**Table 3:** Results (as *P *values) from the validation study genotyping and association analyses for the Caucasian sample, not including GWAS cases and controls.*

		**GWAS Cases and Controls (101 pairs)**	**Caucasians (N = 704)** **(No GWAS cases & controls)**				
					
**SNP**	**Chr**	***P***	**Log(LVMI)**	**LVH**	**MAF**	**Nearest** **object**	**Distance (Mb)**
rs409045	5p13.3	8.47E-07	**0.0101**	0.0798	0.39	*RAI14*	0.028
rs6450415	5q11.2	6.43E-05	0.51	0.98	0.45	*FLJ35954*	0.10
rs1833534	5q34	9.62E-05	0.49	0.54	0.12	*CCNG1*	0.044
rs6961069	7q21	7.34E-05	0.97	0.38	0.41	*SEMA3C*	0.040
rs10499859	7q21	3.27E-06	0.65	0.22	0.45	*CD36*	0.15
rs4129000	12q14.3	2.26E-05	**0.035**	0.18	0.16	*LOC729298*	0.095
rs4129218	12q13.3	5.19E-05	**0.007**	**0.038**	0.21	*LOC729298*	0.10
rs1155635	13q21.33	9.09E-05	0.92	0.57	0.39	*KLHL1*	0.27
rs238688	20p13	8.33E-05	0.31	0.59	0.27	*ATRN*	Intron
rs756529	20q13.13	9.10E-05	0.74	0.32	0.41	*KCNB1*	Intron
							
rs10483186	22q12	3.12E-05	0.55	0.086	0.46	*LOC91464*	0.12

*Analysis adjusted for age, sex, weight, systolic blood pressure, and diabetes; Chr = chromosome; GWAS = genome-wide association study; LVMI = left ventricular mass index defined as LV mass/height^2.7^; LVH = left ventricular hypertrophy defined as LVMI > 47 g/m^2.7 ^in women and > 50 g/m^2.7 ^in men; MAF = Overall frequency of carriers of minor allele (homozygotes + heterozygotes); SNP = single-nucleotide polymorphism

**Table 4:** Results (as *P *values) from the validation study genotyping and association analyses for the Caucasian sample, including GWAS cases and controls.*

		**GWAS Cases and Controls (101 pairs)**	**Caucasians (N = 906)** **(With GWAS cases & controls)**				
					
**SNP**	**Chr**	***P***	**Log(LVMI)**	**LVH**	**MAF**	**Nearest** **Object**	**Distance (Mb)**
rs409045	5p13.3	8.47E-07	**0.0015**	**0.0006**	0.38	*RAI14*	0.028
rs6450415	5q11.2	6.43E-05	0.075	0.068	0.45	*FLJ35954*	0.10
rs1833534	5q34	9.62E-05	**0.0063**	**0.0118**	0.13	*CCNG1*	0.044
rs6961069	7q21	7.34E-05	0.062	0.56	0.41	*SEMA3C*	0.040
rs10499859	7q21	3.27E-06	0.091	0.56	0.45	*CD36*	0.15
rs4129000	12q14.3	2.26E-05	**< 0.0001**	**0.0002**	0.16	*LOC729298*	0.095
rs4129218	12q13.3	5.19E-05	**< 0.0001**	**0.0001**	0.21	*LOC729298*	0.10
rs1155635	13q21.33	9.09E-05	**0.024**	**0.0041**	0.38	*KLHL1*	0.27
rs238688	20p13	8.33E-05	0.56	0.66	0.27	*ATRN*	Intron
rs756529	20q13.13	9.10E-05	0.36	0.18	0.41	*KCNB1*	Intron
							
rs10483186	22q12	3.12E-05	0.065	0.38	0.46	*LOC91464*	0.12

*Analysis adjusted for age, sex, weight, systolic blood pressure, and diabetes; Chr = chromosome; GWAS = genome-wide association study; LVMI = left ventricular mass index defined as LV mass/height^2.7^; LVH = left ventricular hypertrophy defined as LVMI > 47 g/m^2.7 ^in women and > 50 g/m^2.7 ^in men; MAF = Overall frequency of carriers of minor allele (homozygotes + heterozygotes); SNP = single-nucleotide polymorphism

**Table 5:** Results (as *P *values) from the validation study genotyping and association analyses for the African American sample.*

		**GWAS Cases and Controls (101 pairs)**	**African Americans (N = 1467)**				
					
**SNP**	**Chr**	***P***	**Log(LVMI)**	**LVH**	**MAF**	**Nearest** **object**	**Distance (Mb)**
rs409045	5p13.3	8.47E-07	0.096	0.16	0.33	*RAI14*	0.028
rs6450415	5q11.2	6.43E-05	0.44	0.93	0.41	*FLJ35954*	0.10
rs1833534	5q34	9.62E-05	0.67	0.052	0.14	*CCNG1*	0.044
rs6961069	7q21	7.34E-05	0.36	0.55	0.47	*SEMA3C*	0.040
rs10499859	7q21	3.27E-06	0.65	0.69	0.30	*CD36*	0.15
rs4129000	12q14.3	2.26E-05	0.83	0.51	0.21	*LOC729298*	0.095
rs4129218	12q13.3	5.19E-05	0.26	0.17	0.41	*LOC729298*	0.10
rs1155635	13q21.33	9.09E-05	0.53	0.73	0.22	*KLHL1*	0.27
rs238688	20p13	8.33E-05	**0.013**	0.09	0.28	*ATRN*	Intron
rs756529	20q13.13	9.10E-05	**0.048**	**0.04**	0.44	*KCNB1*	Intron
rs10483186	22q12	3.12E-05	0.28	0.63	0.27	*LOC91464*	0.12

*Analysis adjusted for age, sex, weight, systolic blood pressure, and diabetes; Chr = chromosome; GWAS = genome-wide association study; LVMI = left ventricular mass index defined as LV mass/height^2.7^; LVH = left ventricular hypertrophy defined as LVMI > 47 g/m^2.7 ^in women and > 50 g/m^2.7 ^in men; MAF = Overall frequency of carriers of minor allele (homozygotes + heterozygotes); SNP = single-nucleotide polymorphism

## Discussion

Using a GWAS and validation study design, we identified novel SNPs associated with increased LV mass, a phenotype with strong attributable risk for cardiovascular mortality and morbidity. We identified 12 SNPs from the GWAS conducted among cases and controls. The high false discovery rate (i.e., 75% of our SNPs were likely false positives) resulting from our limited sample size in the GWAS was acceptable, as this stage of the research was intended to capture the best pool of signals that could subsequently be tested in the validation study. In the validation study, we were able to successfully genotype 11 of these 12 SNPs in the remaining members of the HyperGEN sample. Among 704 Caucasian participants, 3 of the 11 SNPs were associated with our phenotypes (rs409045, rs4129000, rs4129218). Among 1467 African American HyperGEN participants in the phase two sample, we found modest associations for two of the 11 SNPs (rs238688 and rs756529, chromosome 20).

Of the 11 SNPs that were moved forward to the validation study, two (rs238688 and rs756529, chromosome 20) were intragenic (see Tables 3, 4, and 5). The former is located in the attractin gene (*ATRN*), also known as the mahogany gene because of its role in determining coat color in mice. *ATRN *is involved in diet regulation of obesity in mice and may be relevant for human obesity. This may be important in the current context because body weight is the strongest predictor of LV mass among all factors measured in the HyperGEN study. However, we did not find an association between this SNP and body weight, body mass index, or waist size in HyperGEN. In addition to its potential effects on LV mass and body size, *ATRN *function parallels that of proteoglycan receptors that are responsible for regulating cell interactions during inflammatory reactions. Either of these mechanisms (energy metabolism and/or inflammation) could be important pathways for the development of LV hypertrophy. The second chromosome 20 SNP, rs756529, lies within the potassium voltage-gated channel, Shab-related subfamily, member 1 gene (*KCNB1*). This gene is particularly interesting because its protein product is dephosphorylated by calcineurin. Calcineurin, in both animal and human studies, is associated with LV hypertrophy [18], and this may reflect a unique mechanism for development of LV hypertrophy in hypertension. We have previously shown a strong association between genetic variants in the promoter region of this gene and LV mass in this same population [18].

GWAS genotyping was done only in Caucasians, although in the validation study both the Caucasian and African American families within HyperGEN were genotyped. We observed different association results in Caucasians and African Americans. This lack of a consistent finding between ethnic groups is not unexpected since the allele frequencies were quite different between the two ethnic groups for many of the SNPs, and allele frequencies impact the power to detect association. Many risk factors for LV hypertrophy, including weight and hypertension, also differ between the groups. If the genetic effect estimated by the SNP is influenced by these risk factors, then the association observed would also be different in Caucasians and African Americans.

We compared our findings to Vasan et al.'s GWAS that genotyped 1345 participants from the Framingham Heart Study using the same 100 k GeneChip [34]. Although none of the 12 most promising SNPs from the GWAS were among the top SNPs associated with echocardiographic traits in Vasan et al., two of their most significant SNPs (rs580859 associated with LV diastolic dimension, rs26438 associated with aortic root diameter) were located within 2 Mb of SNPs in our top 12 (rs1833534, rs1155635). Future work within HyperGEN will explore these regions more extensively.

The relatively small sizes of our case and control groups may have been seen – *a priori *– as a limitation of this study. Although it is likely that we missed a number of interesting genetic associations of smaller magnitude, the findings we do have were statistically significant in the validation study. Therefore, *a posteriori *concerns about power are largely academic. In general, small sample sizes are a concern because they increase the chances of false negative findings (i.e., increased Type 2 errors). The extreme degree of multiple testing inherent in GWAS (and the commensurate increase in the harvest of false positives) is explicitly addressed by the GWAS-validation study design. Indeed, five of the 11 SNPs identified during the GWAS were significant in the validation, suggesting that 55% (i.e., 100% minus 45%), were false positive findings, which is much less than predicted by our FDR method calculation (i.e., 75%).

Another limitation of this study is the relative lack of whole-genome coverage afforded by the Affymetrix GeneChip Human Mapping 100 k Set, especially when viewed in the context of more recently developed chips that offer significantly greater coverage. Although it is therefore true that our findings are far from comprehensive, the relatively small net cast by the 100 k chip does not diminish the relevance of the significant associations that we found. An additional concern could also be potential population stratification; however, this is minimized by the family design of our study. We also examined the potential for population structure using a principle components analysis. We identified 6 principle components from the GWAS data; however, adjustment for these did not materially change the results (data not presented).

Although using a sample comprised of only the center of the LVMI distribution for the validation study admits some spectrum bias, this design offers a number of distinct advantages. For example, by evaluating the importance of the SNPs in the middle percentiles of the LVMI distribution, we might better assess the relevance of the variants for the overall hypertensive population. Because our validation study made use of samples from the same source cohort the GWAS cases and controls were drawn from, we eliminate population stratification as a possible explanation of findings. Finally, the phenotypes used in both the GWAS and validation study were collected by the same technicians and read by the same readers, thereby reducing measurement bias as an explanation for any lack of consistent findings.

## Conclusion

In summary, we found suggestive evidence of novel SNPs associated with LV hypertrophy on chromosomes 5 and 12 in Caucasians, and chromosome 20 in African Americans. Future work will characterize these regions with a greater number of SNPs in order to further confirm the genomic regions as true positives and to identify the putative genetic variant and its functional relevance with respect of LV hypertrophy.

## Abbreviations

GWAS: genome-wide association study; LV: left ventricle or left ventricular; LVH: left ventricular hypertrophy; LVMI: left ventricular mass index (defined here as LV mass [in grams] divided by height^2.7 ^[in meters]); SNP: single nucleotide polymorphism; HWE: Hardy-Weinberg equilibrium

## Competing interests

The authors declare that they have no competing interests.

## Authors' contributions

DKA conceived and designed the study, interpreted the data, and drafted the manuscript. NL performed statistical analyses for the GWAS. WT performed statistical analyses for the validation study. DCR conceived and designed the study, offered critical guidance on the biostatistical analyses, and provided a substantive review of the manuscript. RBD conceived and designed the study, collected and warranted the quality of the echocardiographic phenotype data, and provided a substantive review of the manuscript. SAC conducted secondary research and drafted the manuscript. RK produced and warranted the quality of the genetic data. UB conceived and designed the study, guided the work of the genetics laboratory, and provided a substantive review of the manuscript. All authors read and approved the manuscript.

## Pre-publication history

The pre-publication history for this paper can be accessed here:


